# Safety aspects of *de novo* DCB-only PCI—a practical checklist and a simplified revised dissection classification

**DOI:** 10.3389/fcvm.2025.1655201

**Published:** 2025-11-20

**Authors:** Natasha H. Corballis, Ioannis Merinopoulos, Rajkumar Natarajan, Tharusha Gunawardena, Upul Wickramarachchi, Vassilios S. Vassiliou, Antonio Colombo, Bruno Scheller, Simon Eccleshall

**Affiliations:** 1 Department of Cardiology, Norfolk and Norwich University Hospital NHS Foundation Trust, Norwich, United Kingdom; 2 Norwich Medical School, University of East Anglia, Bob Champion Research and Education, Norwich, United Kingdom; 3 Department of Biomedical Sciences, Humanitas University, Milan, Italy; 4 Humanitas Clinical and Research Center IRCCS, Rozzano, Italy; 5 Clinical and Experimental Interventional Cardiology, Saarland University, Saarbrucken, Germany

**Keywords:** drug-coated balloon angioplasty, lesion preparation, coronary dissection, PCI, dissection classification

## Abstract

There is increasing use of drug-coated balloons (DCBs) in *de novo* coronary disease, supported by an ever-expanding evidence base. However, DCB-only angioplasty requires a slightly modified lesion preparation strategy to ensure an optimal angioplasty result and minimise the risk of vessel-threatening dissection. In this article, we discuss the importance of optimal lesion preparation and vessel safety based on clinical and angiographic findings, as well as the selection and deployment of appropriate DCB. We outline a new and simplified classification of dissections: those that are safe to leave untreated (type 1) and those that require modification or stenting (type 2). We also present this classification in a simple graphical format. Finally, we provide a checklist for the complete process. This review article aims to accelerate the learning curve for DCB-only percutaneous coronary intervention (PCI), highlighting the importance of lesion preparation and dissection assessment while ensuring patient safety throughout the procedure. We hope this will facilitate the adoption of safe DCB-only PCI.

## Background

Drug-coated balloon (DCB)-only percutaneous coronary intervention (PCI) is a rapidly expanding area of coronary intervention. However, to protect patients while encouraging increased uptake of appropriate DCB use, it is vital to have a meticulous procedural technique. It is imperative that the early safety of this new stentless PCI approach is well documented. Having developed a learning curve with over 10 years of experience with DCB-only angioplasty at our centre, along with our publications from the SPARTAN registry (1–7), we now delineate our approach to lesion preparation for DCB-only PCI. DCB randomised controlled trials (RCTs) have reported very low acute vessel closure rates (8–11), and we have now confirmed these findings at our centre through a retrospective safety analysis of 10,922 lesions, showing a 0.2% acute vessel closure rate with DCB compared with 0.3% with DES (6). We have developed an approach to managing balloon angioplasty-induced coronary dissections, which is reflected in our safety data. This approach involves optimal lesion preparation to reduce the occurrence of vessel-threatening dissections (VTDs), recognising dissections that are unsafe and subsequently modifying or stenting them. We note that the previous National Heart, Lung, and Blood Institute (NHLBI) dissection classification could perhaps be improved to be more relevant to present-day PCI. Therefore, we propose a new dissection classification and an associated checklist, which we apply to our cases to help operators safely and more quickly adopt this technique into their own PCI practice.

## Lesion preparation for DCB-only PCI

Lesion preparation is a vital component of all PCI, but it is perhaps more important in the setting of a DCB-only approach. It requires a different mindset to maximise the likelihood of completing the procedure safely with a DCB rather than resorting to a bailout DES. The aim is to achieve maximal lumen gain while avoiding excessive vessel trauma with the potential occurrence of VTDs. Our learning curve for such techniques included using lower pressures and increasing the utilisation of specialised balloons and calcium-modification tools as required, as outlined in the following.

The majority of publications (8, 10, 12, 13) rely on angiographic assessment for vessel sizing, adequacy of lumen gain, and safety indicators, while the role of intravascular imaging (IVI) in DCB-only angioplasty remains to be fully defined. As such, we utilise a 1:1 balloon:artery ratio based on angiography for final lesion preparation, following administration of intracoronary glyceryl trinitrate. Our standard approach is to use a non-compliant (NC) balloon because it provides more reliable expansion and is associated with fewer VTDs than semi-compliant (SC) balloons (14). If a less-than-nominal balloon inflation (e.g., 6 atmospheres) allows full balloon expansion, we accept such lower balloon inflation pressures to reduce the risk of VTDs while still obtaining adequate lumen gain. If full balloon expansion is not achieved at low pressures, then gradual and prolonged balloon inflations to nominal or higher pressures are undertaken, similar to the approach used in the early era of balloon angioplasty (15). We try to avoid higher-pressure inflations and the occurrence of dog-boning, as these may otherwise result in VTD (16). The failed expansion of the NC balloon requires careful evaluation and consideration of IVI to understand lesion morphology and may prompt the use of scoring, cutting, intravascular lithotripsy, or other calcium modification techniques as appropriate to ensure adequate lesion preparation.

Up to 30% recoil is acceptable in the setting of previously well-documented full balloon expansion (1:1) and TIMI III flow (12). True recoil is usually best managed using a scoring or cutting balloon in a stepwise approach, similar to our approach of initial balloon dilatation outlined previously. In our experience, scoring or cutting plays a particular role in DCB-only PCI. The focused, longitudinal application of force along the vessel has been shown to deliver better acute gain and reduce the risk of VTDs by creating controlled longitudinal dissections that remain in continuity with the true lumen (17). This technique may also be used to modify a VTD into a safe dissection by fenestrating the contained dissection (and its associated intramural haematoma), restoring continuity to the true lumen (18). Particular attention should be paid to both aorto-ostial and ostial bifurcation lesions due to the increased resistance of the more fibromuscular tissue to simple balloon angioplasty (19). We routinely score or cut such lesions with a 1:1 balloon-to-artery ratio to achieve better lumen gain (20).

## Coronary dissections

Coronary dissections are still defined as per the NHLBI classification from the pre-stent era (21). This system includes six dissection categories, with type A–B dissections considered benign in the setting of balloon-only PCI (12). The classification was based on historical angiographic interpretations from a small number of cases, with fewer good-quality images, more rudimentary equipment, and no dual antiplatelet therapy. We now know, almost by definition, that PCI causes endothelial and atheromatous disruption (22), but only a proportion is visible angiographically, with more injury identifiable using intracoronary imaging. Despite this, the majority of these traumatised vessels are safe and do not require stent implantation on safety grounds (23). Therefore, drawing on the experience of DCB angioplasty in the current era, we have sought to simplify the dissection classification accordingly.

A type A dissection is defined as a minor radiolucent area within the coronary lumen during contrast injection, with little or no persistence of contrast once the dye has cleared (21). This is considered a safe dissection, is probably under-recognised, and likely represents an intimal flap that remains in continuity with the true vessel lumen.

A type B dissection is characterised by a parallel tract or double lumen separated by a radiolucent area during contrast injection, with minimal or no persistence once the dye has cleared (21). There is some ambiguity in this definition, as contrast may persist for more than 1–2 cardiac cycles, but this is then not clearly classified into a different dissection category. Current practice suggests that even if contrast persists for longer than expected, if it is beginning to clear, then it can still be considered a safe dissection. However, significant pooling of contrast or progressive luminal compromise (not due to post-barotrauma spasm) indicates an unsafe dissection. The length of a simple type B dissection should not be a greater cause for concern.

A type C dissection is characterised by contrast outside the coronary lumen (extraluminal cap) with persistence of contrast after the dye has cleared from the lumen and is therefore viewed as a vessel-threatening dissection. However, it is possible to use a scoring or cutting balloon to modify such dissections. Fenestration of the false lumen to re-establish continuity with the true lumen results in a safe dissection (characterised by the absence of persistent contrast).

A type D dissection is a spiral dissection and requires bailout stenting.

Type E is defined as a new, persistent filling defect within the coronary lumen. Although this was historically viewed as a dissection that required bailout stenting, this is no longer concerning. With improved imaging quality, we may recognise type E dissections more frequently; the filling defect may reflect thrombus or merely represent a benign dissection (an intimal flap) viewed in a different plane. Often, further lesion preparation, particularly with scoring or cutting balloons, resolves any ambiguity, and most non-flow-limiting filling defects do not require stent implantation.

A type F dissection involves complete vessel closure and is a clear indication for bailout stenting, but it needs to be differentiated from TIMI 0 flow due to no-reflow.

Therefore, several factors influence the decision on whether a dissection can be safely left untreated:

- Patient's clinical status, as judged by symptoms and ECG, heart rate, and blood pressure changes;
- Maintenance of TIMI 3 flow in the vessel;
- Evidence that contrast is clearing from the dissection planes/vessel wall;
- Absence of progressive luminal compromise;
- Adequate angiographic imaging: This often requires two orthogonal views to safely categorise the dissection. A common error is insufficient acquisition time, which prevents full assessment of contrast clearing. An additional follow-up acquisition without contrast injection can be performed to confirm contrast clearing within 30 s; and
- Vessel tortuosity is relevant because tortuous vessels are more prone to dissection during PCI, more difficult to assess afterwards due to wire artefact, and more difficult to rewire. Acknowledgement of these factors and an awareness of the potential for wire artefact should prompt a very careful assessment of such cases. Withdrawing the wire far enough to allow the vessel to return to a more normal curvature may help reduce artefact.

As our understanding of the safety of leaving dissections has expanded in the DCB-only PCI era (24, 25), we have simplified our classification of dissections as follows:

1. Type 1/non-vessel-threatening dissections—“safe to leave”: These include visible dissections with TIMI III flow and no persistent contrast hang-up. Up to 30% stable luminal compromise, such as that seen with recoil, is considered safe.
2. Type 2/vessel-threatening dissections—“need to stent”: These particularly include dissections with reduced TIMI flow (due to the dissection rather than no-reflow), persistent or accumulating contrast, and/or evidence of progressive lumen compromise due to an accumulating intra-mural haematoma. A spiral dissection remains an indication for stenting.

**Figure 1:**
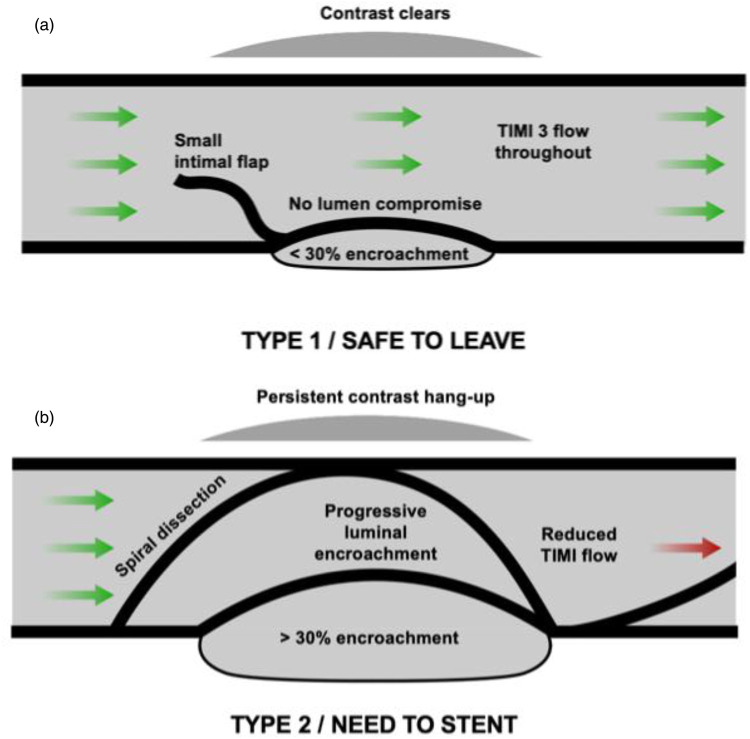
A new and simplified approach to coronary dissection classification. **(a)** Type 1 (safe to leave) dissection. This comprises dissections TIMI 3 flow with the presence of an intimal flap without luminal encroachment or a parallel-tract dissection that appears angiographically outside the vessel wall and from which contrast clears within 30 seconds. **(b)** Type 2 (vessel-threatening/“need to stent”) dissection. This category includes dissections with reduced TIMI flow, dissections that result in progressive luminal encroachment, spiral dissections, or dissections with persistent contrast in a parallel tract that does not clear within 30 seconds.

We adopt an angiography-based approach that defines dissections as either safe (type 1) or vessel-threatening (type 2). The data utilising this approach in our practice have been published, demonstrating excellent safety outcomes with a low acute vessel closure rate of 0.2% (6). These findings need to be interpreted in combination with the list of patient- and lesion-related factors outlined previously. This reclassification of dissections is summarised in Figure 1.

We acknowledge that in certain circumstances, such as complex CTO procedures, different approaches to decisions regarding dissections may apply; however, this lies outside the scope of this paper. Although more complex dissections with residual stenosis may still give good long-term results, the current simplified classification proposed here is recommended for the vast majority of DCB-only *de novo* non-CTO lesions.

We have provided two cases with corresponding images and video files (Supplementary Figures and Videos 1, 2) that highlight both safe and unsafe dissections from our practice.

Case 1 involves a 66-year-old man who presented with transient posterior ST elevation and had no significant medical history. Angiography revealed a culprit ostial circumflex lesion, which was treated, along with bystander disease in the mid-LAD. The mid-LAD lesion was prepared with a 2.5 × 13-mm scoring balloon (NSE Alpha, B. Braun) and then treated with a 2.5 × 15-mm Sequent Please NEO paclitaxel DCB (B. Braun). Subsequent angiography showed a type 1/safe-to-leave dissection with TIMI 3 flow, no significant lumen compromise, and rapid dye clearance.

Case 2 involves a 76-year-old man who presented with a non-ST elevation myocardial infarction. His medical history included hypertension, diabetes, and previous PCI to the RCA and LAD with DES for stable angina. He had a lesion in the RCA just distal to the previous DES. This lesion was prepared with a 3.5 × 30-mm NC balloon and subsequently treated with a 3.5 × 40-mm Sequent Please NEO paclitaxel DCB (B. Braun). There was a type 2/need-to-stent dissection. Although there was TIMI 3 flow and no significant lumen compromise, persistent dye hang-up was evident, particularly in the PA cranial view. This lesion was subsequently stented with a 3 × 38-mm DES and post-dilated to 3.5 mm.

## DCB checklist: assessment prior and following DCB balloon delivery

This outlines a checklist approach to assessing whether a lesion is ready for DCB delivery, focusing on ensuring adequate lesion preparation, a safe angioplasty result/dissection assessment, and allowing deliverability of the DCB. The following factors require assessment:

1. Patient clinical status
  a. Symptoms
  b. ECG findings
  c. Haemodynamic markers of ischaemia
2. Angiographic assessment
  a. Lesion preparation: Ensure that adequate lesion preparation has been performed using intra-coronary vasodilators to optimise vessel size and thus device sizing, confirm that a 1:1 balloon-to-vessel inflation has been achieved with the final pre-dilatation balloon, assess the degree of vessel recoil (usually <30%, signifying an optimal result prior to DCB delivery), and verify that the last—and thus bulkiest—balloon was deliverable, as this serves as a guide for DCB delivery.
  b. Angiographic safety: Identify the presence of any dissection (best done in two orthogonal angiographic views) and use prolonged acquisition to ensure contrast clearance. The presence of a type 1 dissection with TIMI 3 flow indicates a safe result for DCB delivery.
  c. DCB diameter/length decision: Select a DCB with a 1:1 balloon-to-vessel ratio, matching the size of the largest lesion preparation balloon. It is not recommended to upsize the DCB, as it may increase vessel trauma and results in the occurrence of a vessel-threatening Type 2 dissection. The DCB length should be at least 2 mm longer than the pre-dilated vessel segment to avoid geographic miss.
3. DCB time
  a. If there is doubt about DCB deliverability, the use of a guide catheter extension (or perhaps a buddy wire) should be considered to ensure adequate drug delivery and thus good long-term efficacy.
  b. Perform a final fluoroscopic screening to ensure that the guide catheter is adequately engaged and the guidewire is distal enough in the vessel to provide good support prior to DCB insertion into the system, as many DCBs are time-sensitive.
  c. Start a timer when the DCB is introduced to the system to record transit time, ensuring that it remains within the manufacturer's instructions for use (IFU).
  d. Once positioned, the DCB is inflated to nominal pressure for the duration recommended in the device IFU to ensure adequate drug delivery. Warn the patient that prolonged balloon inflation may cause some chest discomfort. The purpose of the DCB is to deliver the drug homogenously to the vessel wall and not to play a role in any further angioplasty; therefore, high inflation pressures are not required.
  e. The same safety checklist should be applied again after DCB delivery, including assessment of patient clinical status, recoil, and dissection.

**Figure 2:**
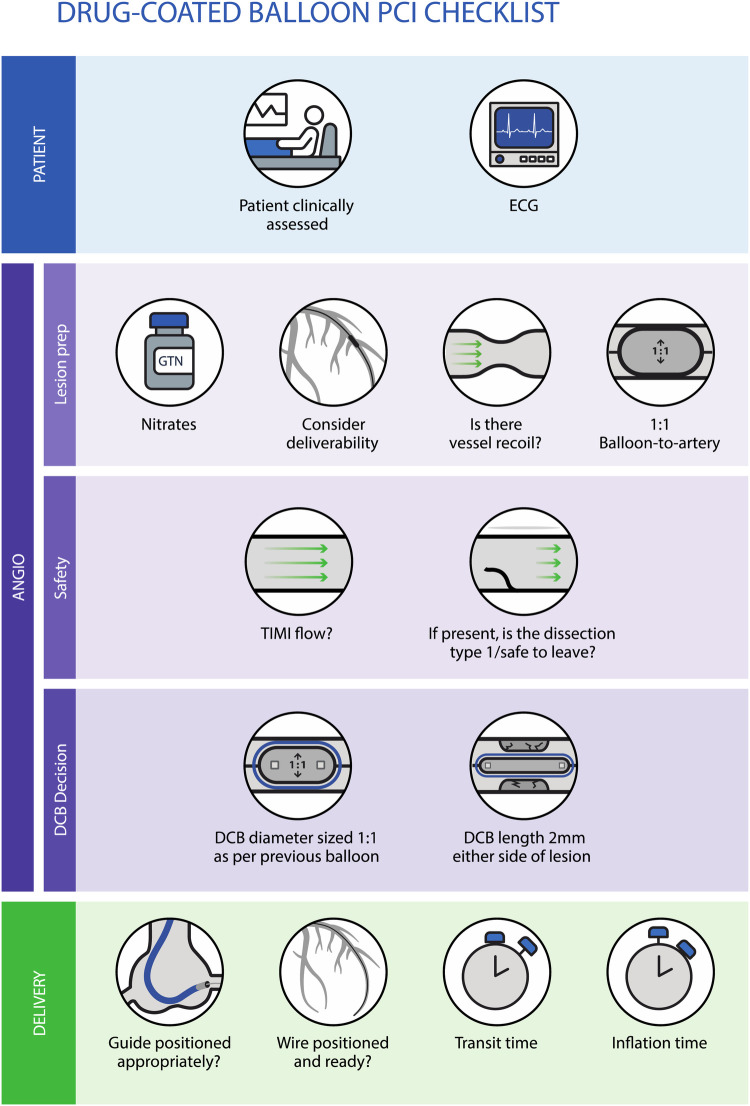
Checklist approach to assess safety and lesion preparation adequacy prior to using a DCB, along with the factors to consider regarding deliverability and sizing of the balloon.

Figure 2 summarises the process for assessing the safety and adequacy of lesion preparation prior to using a DCB, along with the factors to consider regarding deliverability and sizing of the balloon.

## Conclusion

In conclusion, we have outlined our approach to lesion preparation aimed at avoiding a VTD, thereby allowing safe DCB-only angioplasty. We have also sought to improve the current dissection classification to make it clearer in terms of DCB angioplasty classification. Drawing on years of accumulated knowledge and our published safety data on acute vessel closure, we have developed a simplified approach to dissection classification. The majority of dissections are safe to leave untreated, while only a minority of vessel-threatening dissections require a stent. We have also provided our DCB safety checklist. We hope that this simplified classification and checklist will help operators progress through their own learning curves more safely than many of the earliest adopters of this technology.

## Data Availability

The original contributions presented in the study are included in the article/Supplementary Material, further inquiries can be directed to the corresponding author.

## Supplementary material

Supplementary Figure 1 Type 1 dissection/safe to leave. This figure shows a 66-year-old gentleman who underwent PCI to a bystander lesion in the LAD for an acute coronary syndrome. (a) The lesion prior to PCI. (b) Lesion preparation with a 2.5×13 mm NSE alpha. (c) Still image of a type 1 dissection. What becomes apparent in the attached video clip is that this dye clears rapidly with TIMI 3 flow and no significant luminal compromise.

Supplementary Video Case 1 Clip 1 showing the angiogram of the mid-LAD lesion prior to PCI.

Supplementary Video Case 2 Clip 2 showing the delivery of a 2.5×15-mm Sequent Please NEO DCB.

Supplementary Video Case 3 Clip 3 showing a cranial view of a type 1 dissection: there is TIMI 3 flow, no persistent dye hang-up, and no significant luminal compromise.

Supplementary Video Case 4 Clip 4 showing a caudal view of the same lesion, where the dissection is not obviously apparent. Two orthogonal views are important to confirm the apparent safety of a dissection.

Supplementary Figure 2 A 76-year-old man with an NSTEMI undergoing PCI for RCA disease just distal to a previous DES. (a) Culprit lesion. (b) Delivery of a 3.5 × 40-mm Sequent Please NEO DCB. (c) Persistent dye hang-up after the dye has cleared from the remainder of the coronary artery in the LAO view. (d) Persistent dye hang-up in the PA cranial view, with the dye in this view appearing to encircle the lesion. This is a type 2/need-to-stent dissection that was subsequently stented.

Supplementary Video Case 5 Clip 5 showing an angiogram of the lesion prior to treatment.

Supplementary Video Case 6 Clip 6 showing inflation of the DCB.

Supplementary Video Case 7 Persistent dye hang up in LAO view.

Supplementary Video Case 8 Persistent dye hang up in PA cranial view. There is, despite this, TIMI 3 flow and no evidence of luminal compromise, highlighting the importance of long acquisition to ascertain dye clearance.

Supplementary Video Case 9 Clip 9 showing the vessel after stenting and dilatation.
