# Supplementary figures and images for: Safety aspects of de novo DCB-only PCI—a practical checklist and a simplified revised dissection classification
Source: Front Cardiovasc Med. 2025 Nov 20;12:1655201. doi: 10.3389/fcvm.2025.1655201 (PMC12676487; doi:10.3389/fcvm.2025.1655201)

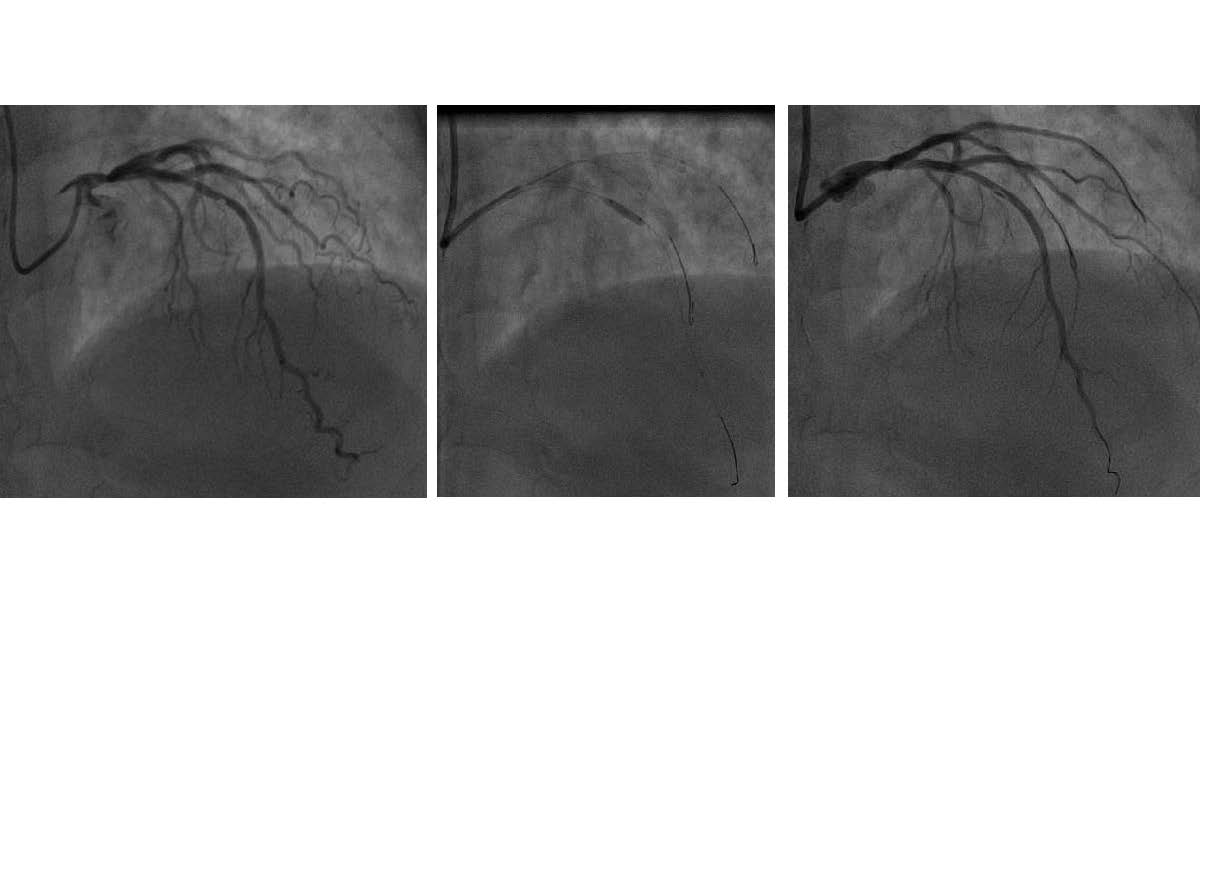

Supplement: Supplementary Figure 1 — Type 1 dissection/safe to leave. This figure shows a 66-year-old gentleman who underwent PCI to a bystander lesion in the LAD for an acute coronary syndrome. (a) The lesion prior to PCI. (b) Lesion preparation with a 2.5×13 mm NSE alpha. (c) Still image of a type 1 dissection. What becomes apparent in the attached video clip is that this dye clears rapidly with TIMI 3 flow and no significant luminal compromise. [file Image1.jpeg]

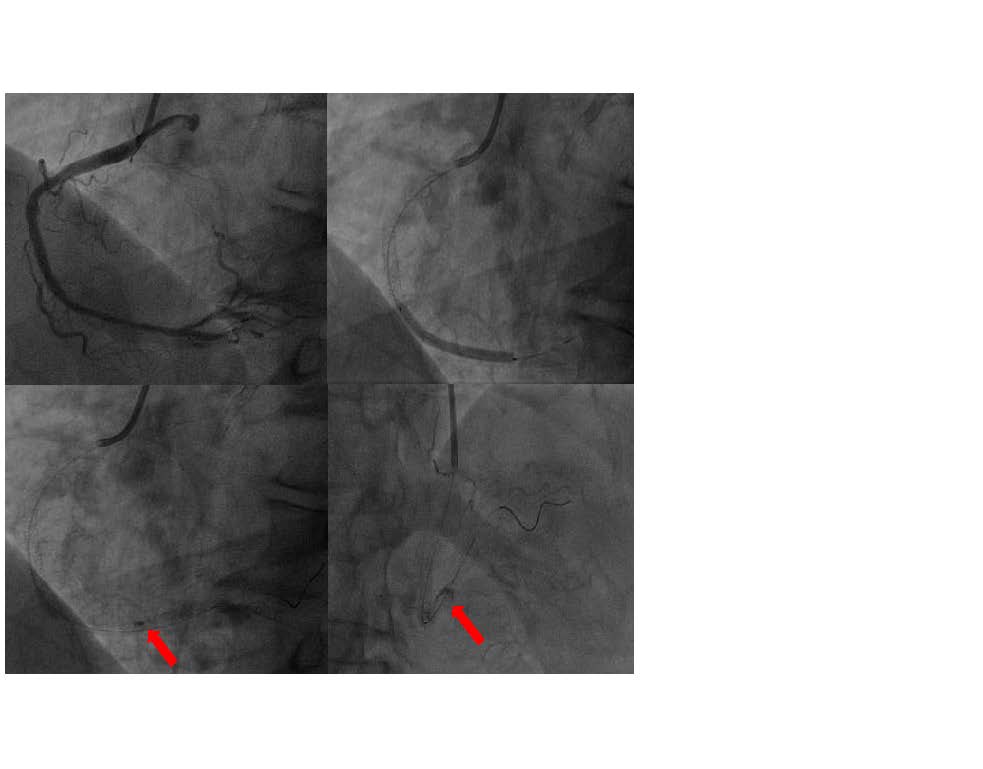

Supplement: Supplementary Figure 2 — A 76-year-old man with an NSTEMI undergoing PCI for RCA disease just distal to a previous DES. (a) Culprit lesion. (b) Delivery of a 3.5 × 40-mm Sequent Please NEO DCB. (c) Persistent dye hang-up after the dye has cleared from the remainder of the coronary artery in the LAO view. (d) Persistent dye hang-up in the PA cranial view, with the dye in this view appearing to encircle the lesion. This is a type 2/need-to-stent dissection that was subsequently stented. [file Image2.jpeg]
